# Pooling financial resources for universal health coverage: options for reform

**DOI:** 10.2471/BLT.19.234153

**Published:** 2019-11-29

**Authors:** Inke Mathauer, Lluis Vinyals Torres, Joseph Kutzin, Melitta Jakab, Kara Hanson

**Affiliations:** a Department of Health Systems Governance and Financing, World Health Organization, avenue Appia 20, 1211 Geneva 27, Switzerland.; b World Health Organization Regional Office for South-East Asia, New Delhi, India.; c World Health Organization Barcelona Office for Health Systems Strengthening, Barcelona, Spain.; d Department of Global Health and Development, London School of Hygiene and Tropical Medicine, London, England.

## Abstract

Universal health coverage (UHC) means that all people can access health services of good quality without experiencing financial hardship. Three health financing functions – revenue raising, pooling of funds and purchasing health services – are vital for UHC. This article focuses on pooling: the accumulation and management of prepaid financial resources. Pooling creates opportunities for redistribution of resources to support equitable access to needed services and greater financial protection even if additional revenues for UHC cannot be raised. However, in many countries pooling arrangements are very fragmented, which create barriers to redistribution. The purpose of this article is to provide an overview of pooling reform options to support countries who are exploring ways to enhance redistribution of funds. We outline four broad types of pooling reforms and discuss their potential and challenges in addressing fragmentation of health financing: (i) shifting to compulsory or automatic coverage for everybody; (ii) merging different pools to increase the number of pool members and the diversity of pool members’ health needs and risks; (iii) cross-subsidization of pools that have members with lower revenues and higher health risks; and (iv) harmonization across pools, such as benefits, payment methods and rates. Countries can combine several reform elements. Whether the potential for redistribution is actually realized through a pooling reform also depends on the alignment of the pooling structure with revenue raising and purchasing arrangements. Finally, the scope for reform is constrained by institutional and political feasibility, and the political economy around pooling reforms needs to be anticipated and managed.

## Résumé

La couverture sanitaire universelle (CSU) consiste à ce que l'ensemble de la population ait accès à des services de santé de qualité sans encourir de difficultés financières. Pour cela, trois fonctions de financement de la santé sont essentielles: le recouvrement des recettes, la mise en commun des fonds et l'achat de services de santé. Cet article s'intéresse à la mise en commun, à savoir le recueil et la gestion de ressources financières prépayées. La mise en commun permet de redistribuer les ressources afin d'offrir un accès équitable aux services nécessaires et d'améliorer la protection financière même en cas d'impossibilité de lever des recettes supplémentaires pour la CSU. Or, dans de nombreux pays, les mécanismes de mise en commun sont très fragmentés, ce qui fait obstacle à la redistribution. Cet article entend donner un aperçu des possibilités de réforme en ce qui concerne la mise en commun afin d'aider les pays qui cherchent à améliorer la redistribution des fonds. Nous présentons quatre grands types de réforme concernant la mise en commun et analysons le potentiel ainsi que les difficultés qu'ils présentent pour mettre un terme à la fragmentation du financement de la santé: (i) passage à une couverture obligatoire ou automatique pour tout le monde; (ii) fusion de différentes caisses afin d'augmenter le nombre de membres d'une même caisse ainsi que la diversité de leurs besoins et de leurs risques; (iii) interfinancement des caisses dont les membres ont des revenus faibles et des risques élevés en matière de santé; et (iv) harmonisation entre les caisses concernant, par exemple, les avantages, les modes de paiement et les tarifs. Les pays peuvent combiner plusieurs éléments de réforme. La réalisation du potentiel de redistribution grâce à une réforme de la mise en commun dépend aussi de l'alignement de la structure de mise en commun sur le recouvrement des recettes et les mécanismes d'achat. Enfin, l'étendue de la réforme est limitée par la faisabilité institutionnelle et politique, et l'économie politique relative à cette réforme de la mise en commun doit être anticipée et gérée.

## Resumen

La cobertura sanitaria universal (CSU) significa que todas las personas pueden acceder a servicios de salud de buena calidad sin experimentar dificultades financieras. Hay tres funciones de financiamiento de la salud que son fundamentales para la CSU: la recaudación de ingresos, la mancomunación de fondos y la compra de servicios de salud. Este artículo se centra en la mancomunación: la acumulación y gestión de recursos financieros prepagados. La mancomunación crea oportunidades para la redistribución de recursos que apoyan el acceso equitativo a los servicios necesarios y una mayor protección financiera, incluso si no se pueden recaudar ingresos adicionales para la CSU. Sin embargo, en muchos países los acuerdos de mancomunación están muy fragmentados, lo que crea barreras a la redistribución. El propósito de este artículo es proporcionar una visión general de las opciones de reforma de la mancomunación para apoyar a los países que están explorando formas de mejorar la redistribución de los fondos. Se describen cuatro grandes tipos de reformas de mancomunación y se discuten sus potencialidades y desafíos para abordar la fragmentación del financiamiento de la salud: (i) pasar a una cobertura obligatoria o automática para todos; (ii) fusionar diferentes fondos para aumentar el número de miembros del fondo y la diversidad de las necesidades y riesgos de salud de los miembros del mismo; (iii) subvención cruzada de fondos que tienen miembros con menores ingresos y mayores riesgos para la salud; y (iv) armonización entre los fondos, tales como beneficios, métodos de pago y tarifas. Los países pueden combinar varios elementos de reforma. La realización efectiva del potencial de redistribución mediante una reforma de la mancomunación depende también de la alineación de la estructura de la mancomunación con los acuerdos de recaudación de ingresos y compra. Por último, el alcance de la reforma se ve limitado por la viabilidad institucional y política, y es preciso anticipar y gestionar la economía política en torno a la reforma de la mancomunación.

## ملخص

تعني التغطية الصحية الشاملة (UHC) أنه يمكن لجميع الأشخاص الحصول على الخدمات الصحية ذات الجودة الجيدة دون مواجهة أزمات مالية. هناك ثلاث وظائف للتمويل الصحي، تعد من الجوانب الحيوية للتغطية الصحية الشاملة، ألا وهي جمع الإيرادات، وتجميع الأموال، وشراء الخدمات الصحية. يركز هذا المقال على التجميع: وهو يعني تجميع الموارد المالية المدفوعة مقدماً وإدارتها. ويؤدي التجميع إلى خلق فرص لإعادة توزيع الموارد لدعم إمكانية الحصول على الخدمات المطلوبة بشكل عادل، مع حماية مالية أكبر حتى لو لم يكن بالإمكان جمع إيرادات إضافية للتغطية الصحية الشاملة. ومع ذلك، فإنه في العديد من البلدان تكون ترتيبات التجميع مجزأة للغاية، وهو ما يؤدي لخلق حواجز في مواجهة إعادة التوزيع. الغرض من هذا المقال هو تقديم نظرة عامة حول خيارات الإصلاح في التجميع، بهدف دعم البلدان التي تستكشف طرقاً لتحسين إعادة توزيع الأموال. سوف نوضح أربعة أنواع عامة لإصلاح التجميع، ونناقش إمكانياتها والتحديات التي تواجه التعامل مع تجزئة التمويل الصحي: (1) التحول إلى التغطية الإجبارية أو التلقائية للجميع؛ و(2) دمج وسائل التجميع المختلفة لزيادة عدد أعضاء هذه الوسائل، وكذلك زيادة التنوع في الاحتياجات والمخاطر الصحية لدى أعضاء التجمع؛ و (3) الدعم المتبادل بين وسائل التجميع التي بها أعضاء من ذوي الإيرادات الأقل والمخاطر الصحية الأعلى؛ و (4) التنسيق بين وسائل التجميع، مثل المزايا وطرق الدفع والمعدلات. يمكن للبلدان الجمع بين عدة عناصر للإصلاح. إن إدراك وجود إمكانية لإعادة التوزيع تتحقق بالفعل من خلال إصلاح التجميع، إنما يعتمد أيضاً على التنسيق بين نظام التجميع، وترتيبات جمع الإيرادات والشراء. وفي النهاية، فإن نطاق الإصلاح يتقيد بالجدوى المؤسسية والسياسية، كما أن الاقتصاد السياسي المرتبط بإصلاح التجميع يحتاج إلى التوقع والإدارة.

## 摘要

全民健康覆盖 (UHC) 意味着所有人都可以享受优质医疗服务，同时不会遇到财务困难。三项医疗融资职能——提高收入、统筹资金和购买医疗服务——对于 UHC 至关重要。本文着重于统筹：积累和管理预付资金。统筹能够创造资源再分配的机会，有助于人人都能公平地享有所需的服务并且得到更大的财务保障。即使无法为 UHC 增加收入，依然会有保障。然而，在许多国家中，统筹安排非常分散，从而对再分配造成阻碍。本文的目的旨在简要概述统筹改革方案，以支持正在探索加强资金再分配方法的国家。我们概述四种类型的统筹改革并讨论其潜力以及在解决医疗融资分散问题时遇到的挑战：(i) 转为强制性或自主性全民覆盖；(ii) 合并不同的统筹方案以增加参保会员人数，同时提升参保会员的医疗需求和风险多样性；(iii) 对于收入较低和健康风险较高的会员参加的统筹方案进行交叉补贴；(iv) 协调各种统筹方案，例如福利、支付方式和费率。各国可以结合多种改革要素。是否能够真正通过统筹改革发挥再分配的潜力还取决于协调统筹结构与提高收入和购买安排。最后，改革的范围因体制和政治上的可行性而受到限制,需要围绕统筹改革进行政治经济学方面的预测和管理。

## Резюме

Всеобщий охват услугами здравоохранения (ВОУЗ) означает, что все люди могут получить доступ к качественным медицинским услугам, не испытывая финансовых трудностей. Три функции финансирования здравоохранения (сбор доходов, объединение средств и приобретение медицинских услуг) жизненно необходимы для достижения всеобщего охвата услугами здравоохранения. Данная статья посвящена вопросу объединения средств — накоплению предоплаченных финансовых ресурсов и управлению ими. Объединение средств создает возможность перераспределения ресурсов для обеспечения равноправного доступа к необходимым услугам и дополнительной финансовой защиты, даже если дополнительные доходы для достижения всеобщего охвата услугами здравоохранения не могут быть собраны. Однако во многих странах механизмы объединения средств часто разрознены, что создает барьеры для перераспределения. Цель данной статьи — предоставить обзор вариантов реформы объединения средств с целью оказания поддержки странам, которые ищут возможности совершенствования механизмов перераспределения средств. Авторы выделяют четыре основных типа реформ объединения средств и обсуждают их потенциальные возможности и трудности в решении проблемы разрозненности финансирования здравоохранения: (i) переход на обязательный или автоматический охват услугами для всех категорий населения; (ii) объединение различных финансовых ресурсов для увеличения количества участников пула средств и разнообразия потребностей и рисков для здоровья среди участников пула; (iii) перекрестное субсидирование пулов, участники которых имеют более низкие доходы и более высокие риски для здоровья; (iv) согласование льгот, способов оплаты и ставок во всей системе пулов средств. Страны могут объединять несколько элементов реформ. Реализация потенциальных возможностей перераспределения финансовых средств в результате реформы объединения средств также зависит от согласования структуры пула с механизмами сбора доходов и закупок. Необходимо отметить, что масштабы реформы ограничены институциональными и политическими возможностями, а политико-экономическая ситуация вокруг реформы объединения средств должна быть прогнозируемой и управляемой.

## Introduction

Universal health coverage (UHC) means that all people can access health services of good quality without experiencing financial hardship, with the objectives of equitable access in line with their needs, and financial protection and fair financing. The way that resources are raised, pooled and used to purchase health services determines whether resources are available at the point of need. Health financing is therefore key to achieving the objectives of UHC. Yet many countries struggle to progress towards UHC due to numerous deficits in raising revenue, pooling funds and purchasing health services. While these three health financing functions are strongly related, this article focuses on the function of pooling.

Pooling is the accumulation and management of prepaid financial resources on behalf of some or all of the population. Out-of-pocket payments are non-prepaid, unpooled funds and thus work against the objectives of UHC.^1^ Pooling is an enabling function, creating opportunities for efficient redistribution of resources to support equitable access to needed services, with financial protection from any given level of prepaid funding. However, pooling is fragmented in many countries, which creates barriers to redistribution and results in inefficiencies.^1^^–3^ A key policy question then is how a country can reform its pooling arrangements to increase redistribution at the system level and across different pools so there is progress towards UHC.

There has been a lack of conceptual work on this subject in the literature since publication of the World Health Report 2010.^1^ Readers can consult other sources for a review of pooling reforms in former communist countries^4^ and for a typology of pooling arrangements.^3^ However, we have not identified any global overview or discussion of pooling reforms from a system perspective. This gap may be due to insufficient recognition that pooling is a distinct health financing policy instrument that can improve financial protection and equitable access to health care, even if additional revenues cannot be raised.

In this article we provide an overview of various options for pooling reforms and assess their potential to increase countries’ capacity to redistribute resources equitably. The aim is to support countries in exploring their pooling options for UHC. We based the article on a review of country experiences in the published and grey literature using the terms “pooling reforms” and “fragmentation in pooling” in a search of the online databases PubMed® and Google Scholar. We supplemented the literature review with insights and information gathered from our policy advisory and technical work on health financing in countries around the world.

## Objectives of pooling

Pooling serves to spread the financial risk associated with the need to use and pay for health services, so that this risk is not fully borne by an individual who falls ill; this is often referred to as risk pooling.^5^ Importantly, risk pooling can be achieved by more than just health insurance, and there are many ways to structure pooling.^1^^,3^

Redistributive capacity refers to the potential to redistribute funds from individuals with lower health needs and lower health risks to individuals with higher health need and risks (health risks meaning the risk of incurring health expenditure). The central objective of pooling is to maximize redistributive capacity by de-linking contributions, such as taxes or insurance premiums, from a person’s health status or health risks.^1^^,^^6^ To achieve these objectives, desirable attributes of a pool of health funds and health risks are size (in terms of the number of people in the pool) and diversity (of health risks within the pool). An important feature of any pool is compulsory or automatic coverage to increase pool size and diversity.^1^ Otherwise the problem of adverse selection may arise, that is, the tendency for individuals with greater health needs to join a voluntary scheme, leading to an imbalance of risks in that pool and limited ability to share risks across people with different health needs.^7^ In the case of multiple pools, the average per capita expenditure on health, adjusted for the pool members’ health risks, should be equal or similar across pools.

When pooling arrangements are fragmented, however, redistributive capacity becomes limited. Fragmented pooling is characterized by differences in people’s health risks across pools, such that the pools with higher health risks need more resources for their pool members to get the services they need. If not matched by greater revenue, this fragmentation can lead to coverage gaps, inequitable access to services and lower financial protection. Fragmented pooling also contributes to health system inefficiencies, due to duplication of tasks, resulting in higher health system administration costs overall.^2^^,^^6^

## Pooling reform options

**Fig. 1:**
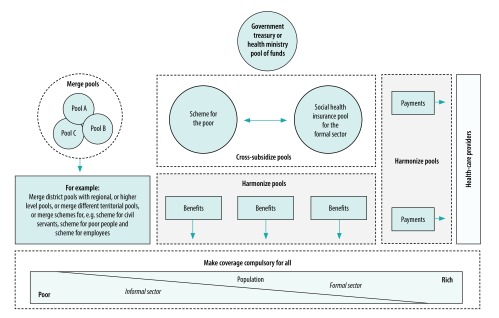
Illustration of pooling reforms for universal health coverage

Notes: Circles refer to a pool; dotted lines refer to a specific pooling reform.

In this section we outline four principle ways of reforming pooling arrangements. These strategies are not mutually exclusive, and countries can combine several elements of them. Fig. 1 provides a visualization of the pooling options, while Table 1 summarizes their features and effects.

**Table 1:** Pooling reforms for universal health coverage: effects and requirements

Type of pooling reform	Effects on the pooling structure	Effects on pooling objectives	Requirements
Making coverage compulsory or automatic	Increases size and diversity of pool	Improves redistributive capacity and efficiency	If contributory: subsidization of those people unable to contribute
Merging pools	Increases size and diversity of pool	Improves redistributive capacity and efficiency	
Cross-subsidization	Maintains the pooling structure	Attempts to equalize available per capita funds across pools	
Harmonization across pools	Maintains the pooling structure	Aligns pool operations and attempts to equalize benefits and conditions at the point-of-service use	

### Making coverage compulsory or automatic

Whatever the pooling structure, a fundamental requirement for increasing a country’s redistributive capacity is to make coverage compulsory or automatic.^1^ Compulsory coverage goes together with contributory-based entitlement; that is, there must be a specific contribution by or on behalf of the covered person. Automatic coverage means that a person is covered based on her residence or citizenship.^8^ When coverage is compulsory or automatic for all population groups, the pool size increases and the pool(s) have a more diverse mix of health risks among their members, since people at all levels of health risk (high and low) are covered.

Some low- and middle-income countries have introduced contributory compulsory coverage for people in the informal economy. These countries manage to enforce this because contributions are highly subsidized by government funding. Thus, all or a large part of the population has the same coverage, with some population groups being fully subsidized, as is the case in Chile, Mongolia and Rwanda, for example.^9^^–^^11^ In other countries, such as Ghana and Viet Nam^12^^,^^13^ there remains a missing middle segment of people who are outside the formal sector, but not considered as poor or vulnerable, and are hence ineligible for fully subsidized schemes. In this case, even when enrolment is officially mandatory, enforcing it is difficult, and this missing middle group may not enrol in the contributory scheme, even if contributions are partially subsidized.^14^^–^^16^ Gaps in automatic or compulsory coverage mean that population groups who are not covered are likely to have higher out-of-pocket expenditure, with the ensuing financial burden resulting in lower use of services.^12^^,^^17^

In summary, merely introducing compulsory or automatic coverage can be both unfeasible and insufficient on its own, as it needs to be accompanied by subsidies for those who cannot contribute, as discussed below.

### Merging pools

One direct way of reducing fragmentation in health financing systems is to decrease the number of pools through merging them. This increases the pool size and the diversity of health risks of the pool(s), thus enhancing redistributive capacity. Moreover, the merging of pools reduces administrative costs because duplication of tasks is reduced. Merging may also enhance the purchasing power of the pool and hence the potential to purchase health services more strategically for gains in efficiency and equity.^2^ Merging can be a solution to various forms of fragmentation.

First, merging can be appropriate when there are too many territorially-based health pools. For example, there may be a pool for each province under general tax financing arrangements where the government administration is decentralized. Merging can occur as part of broader reforms that go beyond the health sector, such as public administration and recentralization reforms. A successful example of such a territorial merger reform is Denmark, which reduced the number of administrative regions from 14 to 5 and of municipalities from 271 to 98, and in doing so lowered the number of health financing pools. This reform helped increase redistributive capacity, strengthen the purchasing power of the pools and save administrative costs.^18^ In other instances, decentralized funds and pools for the health sector only are merged. For example, Ukraine reversed previous budget allocations to lower government levels and instead established a general tax-funded national pooling and purchasing agency.^4^

Second, merging may help in such decentralized health-care systems where there is an additional layer of fragmentation due to territorial overlap of pools. This fragmentation happens when lower levels of government pool and allocate resources to their health-care facilities in their own area, such as district governments to district facilities, and regional governments to regional facilities. In this setup, pooling, purchasing and service provision is vertically integrated, and in principle, there are distinctions in the level of health services to be provided by different levels of facilities. In practice, however, there are overlaps, since districts exist within regions or provinces. Overlapping pools can lead to duplication of infrastructure and inefficient networks for health-service delivery. This issue is particularly evident in provincial capitals, as the provincially funded facilities also provide lower-level services. Not only does this duplication affect efficiency directly, but it also reduces redistributive capacity for a given level of available funds.^4^ Various countries, such as Kyrgyzstan, the Republic of Moldova and Ukraine^4^^,^^19^ have addressed this fragmentation and overlap issue through vertical merging; that is, elevating the level of pooling to higher levels of government. However, this type of pooling reform also implies introducing changes to the service delivery organization and public financial management rules.

Third, pools characterised by population segmentation can be merged. As an explicit policy instrument, this is particularly relevant for many low- and middle-income countries. Such horizontal merging can be applied to two or more health coverage schemes, particularly when characterized by population segmentation. Several countries have taken an explicit policy decision to merge different schemes for different population groups. As such a (previously separate) subsidized scheme for lower income and vulnerable people, who tend to have higher health risks, is combined and integrated with a larger existing scheme for contributory members. Instead of calling it merging, policy-makers may also refer to this as adding or integrating new population groups into the existing (contributory) scheme. Either way, this change usually implies a diversification of the sources of funds to be pooled because (additional) budget transfers are required to fund coverage for those unable to contribute. The aim is to provide the same benefit package for everyone.

Better-off population groups may oppose the merging of pools for fear of having to cross-subsidize poorer groups. Nonetheless, several countries have managed to introduce such reforms, including Indonesia (2014),^20^ the Republic of Korea (2003),^21^ Turkey (2012)^22^ and Viet Nam (2001).^23^ In all countries, the merging of pools significantly increased the risk diversity in the merged pool and was the starting point for reducing inequities in access to health services. In practice, merging of pools and funds can also lead to undesirable effects and increase inequities. In some instances, state budget transfers to finance the coverage of poor and vulnerable population groups did not benefit these groups, but instead cross-subsidized better-off groups.^24^ This outcome is because the better-off groups use health-care services more, and use more expensive services, benefiting from better service availability and geographical access in urban and higher-income areas. While such a merger leads to a higher level of risk sharing, it does not automatically lead to increased spending on the poorer population group. The merger may perpetuate pro-rich spending, particularly when purchasing arrangements undermine the redistributive capacity created by the pooling arrangement, as has been the case in Indonesia and Viet Nam for example.^20^^,^^23^

### Cross-subsidization

When there are multiple pools, an alternative to merging is explicit cross-subsidization through risk adjustment; that is, adjusting pool funding according to the members’ health needs and risks. This option retains the number and structure of multiple pools, and instead redistributes funds with the aim of reaching equal per capita average revenues across pools, adjusted for pool members’ health risks. There are various approaches to adjustment, but common to all of them is that a central pool, or a central-level fund holder, exists or is created in a virtual account. Funds from this central pool are allocated among pools, such as territorially distinct health funding pools, based on an allocation formula.^1^ This mechanism is used in numerous countries with a decentralized system, such as Spain and England in the United Kingdom of Great Britain and Northern Ireland. In these countries, average per capita spending, when risk-adjusted, is similar across the different territories.^25^^,^^26^ The adjustment mechanism may be applied jointly for several sectors, not only for health.^1^ Likewise, funds can flow from the central or virtual pool to different health coverage schemes characterized by population segmentation, as is the case in Japan,^27^ or to competing health insurance funds, such as in Czechia, Germany and Switzerland.^4^^,^^28^^,^^29^ In fact, it is only through risk adjustment that competition among health insurance funds, and hence patient choice of pools, can be realized, as well as a benefit package that is the same for all across pools.

Adjustment for the pool members’ health risks is typically based on assessing the relative health risks of members in that pool, using criteria such as age and sex, employment status, disability and morbidity as well as poverty levels of a region.^30^ The allocation formula can also consider the revenue-raising capacity of the different pools. Risk adjustment enhances redistribution of funds, but it creates an extra administrative burden compared with having a single pool, potentially leading to higher administrative costs. Risk adjustment also requires data and an effective information management system. Nonetheless, in some contexts, introducing risk adjustment mechanisms may be politically more acceptable than merging pools, especially when the political autonomy of different territories is critical, such as in Spain. Moreover, risk adjustment on its own is not enough. Aligning and adjusting the operation and design features of the different pools is also needed, so that they operate in a uniform or at least similar way.

Another form of cross-subsidization is to introduce and subsidize a new pool, especially when setting up a unified pool for different population groups is unfeasible. The idea is to create an explicit non-contributory coverage scheme for people outside the formal sector. Redistribution is achieved by providing budget transfers and gradually increasing these, with the ultimate aim of achieving equitable access to health services and harmonized benefit packages. Countries that have pursued this pooling reform option include for example Colombia,^31^ Gabon,^32^ Mexico,^33^ Peru^6^ and Thailand.^34^ In these countries, reforms have substantially reduced the differences in per capita expenditure between different population groups, and thus helped to improve financial protection and equitable access to health services. To be effective, a new scheme for non-contributory population groups must introduce automatic coverage, whereby all people outside employment in the formal sector are covered, although this automatic coverage has not been the case in all countries using this reform approach.

### Harmonization across pools

The objectives of pooling can also be achieved through policy instruments that go beyond the realm of pooling. Such reform efforts can focus on harmonization across pools, which can be considered an as-if-pooling mechanism. Key areas for harmonization and standardization include the benefit package, contracting arrangements, provider payment mechanisms and remuneration rates, as well as information management systems. For example, in Colombia, benefits were effectively harmonized for the contributory and subsidized schemes, although this reform took several years,^31^ since this requires the same (health-risk adjusted) per capita level of funding. Such harmonization attempts are also currently underway in India,^35^ in addition to its core reform of providing budget transfers to a separate coverage scheme for the poor.

## Policy issues and lessons

Reforming the way in which funds for health are pooled primarily addresses the structure and nature of pooling and is essential for enhanced redistributive capacity. When participation in a health coverage scheme is contributory, subsidization will be needed for certain population groups. In determining which pooling reform option is appropriate, countries need to be clear about the multiple causes of fragmentation in their financing system and use this understanding to define their reform goals and directions.

Whether the potential of pooling reforms is actually realized will also depend on alignment of the pooling structure with the other health financing functions of revenue raising and purchasing. Revenue-raising policies determine the prepaid share of health expenditure and whether funds are raised equitably. Likewise, redistribution only succeeds through appropriate arrangements for purchasing health services to achieve efficiency, equity and financial protection objectives. These arrangements include setting suitable and coherent incentives for providers to deliver quality health-care services.^36^ Importantly, provider payment methods and amounts of payments to health-care providers should be the same for all members of the pool, independent of whether people pay direct contributions or not.

Misalignment of pooling and purchasing arrangements is also common in universal tax-funded systems in which the health budget is the dominant pooling arrangement. Misalignment may happen when the budget is allocated to providers based on historically set budget lines that are determined by an input logic, that is, how much inputs (such as staff, medicines and supplies) are needed (rather than paying for the output, such as services provided or patient cases treated).^37^ Budget allocations for vertical disease programmes may also result in misalignment. Addressing these shortcomings will be an important step towards realizing the potential of a health budget as a unified pool. Moreover, pooling reforms may also require changes in public financial management procedures, including how budgets are formulated and implemented.

In many countries, the source of funds for health is still associated with a pooling arrangement. However, there is no inherent link between how resources are raised and how they should be pooled. Diverse sources of revenues can be combined in a pool before these funds are passed on to providers. Therefore, delinking sources of funds from pooling options is important. 

The question has been raised whether non-contributory coverage for those outside the formal sector could encourage informalization of the labour force, that is, an increase in the share of people working in the informal economy. Evidence is scarce and mixed. For example, the effect of Mexico’s reforms was marginal; the proportion of the population in the formal sector decreased by 0.4–0.7 percentage points within a few years of the programme’s introduction.^38^ In contrast, in Thailand informal-sector employment increased by two percentage points in the year of adopting universal coverage and just under 10 percentage points after three years.^39^ However, people need access to health services and financial protection immediately. The objective of UHC cannot be traded against the need to expand formal employment, which requires other policy instruments and is a long-term economic policy goal.

Finally, as changes in pooling arrangements are about redistribution of funds, it is important to recognize that there may be institutional and political constraints on the scope for action to reduce fragmentation in a health financing system or to mitigate its consequences. Reform requires the time and institutional capacity to implement it, as well as the approval of decision-makers and involved stakeholders. Clearly, pooling reforms go beyond the realm of health ministries and require strong support from other government agencies. Despite the complexities of political economy, we urge countries to undertake pooling reforms.

In conclusion, a variety of pooling reform options are available to enhance redistribution of resources for health. For such reforms to realize their potential, however, they must be set within an overall vision of health financing that aligns pooling with other health financing functions.
